# Understanding the Pathways of Neighborhood Environmental Influences on Health Among U.S. Chinese Older Immigrants

**DOI:** 10.1093/geroni/igaa057.364

**Published:** 2020-12-16

**Authors:** Yi Wang, Man Guo, Jinyu Liu, Kara Carter

**Affiliations:** 1 University of Iowa, Iowa City, Iowa, United States; 2 Weatherhead East Asian Institute, New York City, New York, United States

## Abstract

Neighborhood environment has proven to be consequential for older Americans’ physical, mental and cognitive health. However, this topic is much less studied among older Asian immigrants, a fast growing immigrant group who embrace values of collectivism and community connections. The current study used the first wave data (N=2920) of the Population Study of Chinese Elderly (PINE), the largest population-based sample of Chinese older adults in the U.S., 1) to examine the direct associations between neighborhood environment (social cohesion, physical disorder) and health outcomes (self-rated health, depression, and cognitive health); and 2) to identify possible mediators at intrapersonal (sense of hopelessness, sense of mastery) and interpersonal levels (social engagement, cognitive engagement) through which neighborhood environment influences health. The results of Sobel tests from path analysis showed that neighborhood social cohesion was associated with better health outcomes on all the domains: self-rated health (b= 0.050, p<.01), depression (b= -0.202 p<.001), and cognitive health (b=0.092, p<.001), whereas neighborhood physical disorder was associated with poorer self-rated health (b= -0.069, p<.01) and more depressive symptoms (b=0.174, p<.001). Full and partial mediations were detected. For example, neighborhood physical disorder influences depression completely through intrapersonal traits, higher sense of hopelessness (b=1.879, p<0.001) and reduced sense of mastery (b= -2.656, p<0.001). Neighborhood social cohesion contributes to better cognitive health partially through increased social engagement (b=1.696, p<0.001) as well as cognitive activities (b=1.392, p<0.001). The findings identified the ecological component in resilience building processes, and provide evidence for mezzo-level intervention to improve health among aging U.S. Chinese immigrants.

